# Atypical anti-GQ1b antibody syndrome presenting with vomiting as the initial symptom: a case report and literature review

**DOI:** 10.1186/s12883-023-03213-7

**Published:** 2023-04-27

**Authors:** Jie Deng, Lei Wu, Songqing Wei, Xiaofo Zhang

**Affiliations:** The Affiliated Changsha Central Hospital, Department of Pediatrics, Hengyang Medical School,University of South China, Changsha, China

**Keywords:** Anti-GQ1b antibody syndrome, Vomiting, Abnormal gait, Case report

## Abstract

**Background:**

Anti-GQ1b antibody syndrome is a rare autoimmune neuropathy, and atypical cases are even more rare, only a few cases have been reported. Anti-GQ1b antibody syndrome is difficult in early diagnosis and prone to misdiagnosis. Generally,in children with anti-GQ1b antibody syndrome,extraocular muscle paralysis is the initial symptom. However, anti-GQ1b antibody syndrome with vomiting as the initial symptom followed by abnormal gait has not been reported.

**Case presentation:**

We reported a case of anti-GQ1b antibody syndrome with vomiting as the initial symptom, followed by abnormal gait. One day after vomiting, the child developed abnormal gait, which primarily manifested as a slight tilt of the upper body during walking as well as an opening and swaying of the legs at fast walking paces,then progressively aggravated, and finally he could not stand on his own.In the auxiliary examination, cerebrospinal fluid routine,biochemical and metagenomic Next-Generation Sequencing (DNA and RNA), brain + spinal cord contrast magnetic resonance imaging (MRI),magnetic Resonance angiography (MRA) and diffusion-weighted image (DWI), hip and knee joint ultrasound showed normal results. Anti-GQ1b antibody syndrome was not confirmed until the positive anti-GQ1b IgG antibody was detected in the serum. After treatment with intravenous immunoglobulin (IVIG) and glucocorticoid, the child recovered well, and a 3-month outpatient follow-up showed that the child was able to walk normally.

**Conclusions:**

There are no previous reports of anti-GQ1b antibody syndrome with vomiting as the initial symptom, followed by abnormal gait. Therefore, this valuable case contributes to expanding the database of clinical manifestation of anti-GQ1b antibody syndrome, so as to improve pediatricians’ awareness about such rare diseases and reduce misdiagnosis.

## Background

Anti-GQ1b antibody syndrome is a type of neurological disease related to autoimmunity. The detection of anti-GQ1b-IgG antibody in the serum of patients is highly disease-specific and is the basis for the diagnosis of this syndrome [1]. The incidence rate of this kind of disease is low, and there are only case reports at present. It is a rare disease in autoimmune diseases and atypical cases are even rarer,which make early diagnosis in patients difficult and easily lead to misdiagnosis. Anti-GQ1b antibody syndrome has a variety of clinical manifestations, mainly manifested as acute eye muscle paralysis, disturbance of consciousness, weakened or absent tendon reflexes, and clinical phenotypes include Miller-Fisher syndrome (MFS), acute ophthalmoparesis (AO), Bickerstaff’s brainstem encephalitis (BBE), pharynx-cervical-brachial weakness (PCB), FS plus Guillain-Barré Syndrome and atypical anti-GQ1b antibody syndrome [2]. Unlike MFS, which is the most common clinical phenotype in adults [3], AO and BBE are the most common clinical phenotypes of anti-GQ1b antibody syndrome in children, and most of them have external ophthalmoplegia as the initial clinical symptom [4]. This paper reports a case of atypical anti-GQ1b antibody syndrome in a child with vomiting as the initial symptom followed by abnormal gait. To our knowledge,this is the first case report so far. In addition,we summarized the clinical characteristics of anti-GQ1b antibody syndrome in children combined with the literature data, aiming to improve pediatricians’ awareness about this rare disease.

## Case presentation

An 8-year-old male child was admitted to our hospital with the chief complaint of “vomiting for 2 days, weakness of lower limbs and abnormal gait for 1 day”. Six days before illness onset,he had a history of sore throat and fever.Two days before presenting at the hospital, He began to vomit.The next day of vomit he felt weak in his lower limbs and had abnormal gait. He had no cough, abdominal pain, diarrhea, limb joint pain, chest pain and other discomforts. After admission, dizziness appeared,but no vertigo, continuous vomiting, more than 10 times/day, with a little coffee-colored liquid can be seen when vomiting is severe.Abnormal gait primarily manifested as a slight tilt of the upper body during walking as well as an opening and swaying of the legs at fast walking paces, then progressed to inability to walk, and eventually have difficulty standing on his own. No spontaneous or position-induced nystagmus, no external ophthalmoplegia, clear speech, no dysphagia, or incontinence. Past history, growth and development history, family history (-). Physical examination: clear consciousness, poor mental state, cardiopulmonary and abdominal systems (-). Cranial nerve examination (-), limb muscle strength level V, muscular tension normal, finger-to-nose test, romberg test(+), heel-knee-shin test left side is slightly less accurate than right side, rapid alternating movements test(-), Deep and shallow sensation is normal, bilateral biceps tendon reflex, triceps tendon reflex and brachioradialis tendon reflex (2+), bilateral patellar tendon reflex and achilles tendon reflex (+), right Babinski’s sign is suspiciously positive, meningeal irritation Sign negative.

Laboratory data: blood, urine and stool routine, blood biochemistry, erythrocyte sedimentation rate, coagulation, cholinesterase, blood ammonia, ceruloplasmin, methionine, complement C3 and C4, blood tumor markers, vitamin D value, folic acid(-); immunoglobulin A 2.31 g/L (normal range,0.33 ~ 1.78); immunoglobulin G 13.90 g/L (normal range,5.96 ~ 13.64); On days 2 and 6 of admission, cerebrospinal fluid routine, biochemical and metagenomic Next-Generation Sequencing (DNA and RNA) (-), and normal opening pressure of 140mmH2O; blood and cerebrospinal fluid autoimmune brain antibody IgG (NMDAR, AMPAR1, AMPAR2, LGI1, CASPR2, GABABR), cerebellar antibody profile Seven Antibody IgG (GAD65, Tr(DNER), Zic4, CV2 (CRMP5), Homer3, ATP1A3, ARHGAP26) and anti-MOG antibody IgG were negative.On the sixth days of admission,the serum anti-GQ1b antibody IgG was positive, and the GD1b and GM1 antibodies were negative (the above anti-ganglioside antibody detection was completed by the Dot Immunobinding Assay of a third-party company). Audiometry and otoscopy were normal. Abdominal + hip and knee joint ultrasound (-), head CT (-), On days 2 and 10 of admission, brain + spinal cord contrast magnetic resonance imaging (MRI),magnetic Resonance angiography (MRA)and diffusion-weighted image (DWI)(-); Electro encephalograph(EEG) showed that a small amount of sharp-wave-like waves can be seen in the right parietal occipital lobe.Additionally,the nerve electromyography revealed that there was a decrease in the frequency and an abnormal waveform of F wave in the motor nerves of both upper limbs, which fell inside the normal range of latency. However,there was no obvious abnormality in peripheral nerve conduction for the patients’limbs, F wave and H reflex of both lower limbs.

After admission, firstly, he was given omeprazole stomach protection and fluid rehydration and other symptomatic treatment, but the condition did not improved, with a progressive deterioration.On day 4 of admission,the patient was given intravenous immunoglobulin (IVIG) therapy (400 mg/kg/day for 5 days).After 2 days of IVIg treatment, the child still vomited frequently and could not stand on his own.On day 6 of admission,intravenous methylprednisolone(20 mg/kg for 3 days, 10 mg/kg for 3 days) was added for therapy. Then the child’s vomiting and dizziness were relieved and he recovered from being unable to stand on his own to being able to support himself. After walking a few steps, the intravenous methylprednisolone was stopped and changed to oral prednisone acetate tablets, and the dose was gradually reduced. After 3 months, the outpatient follow-up showed that the child returned to normal walking.

## Discussion and conclusions

The concept of anti-GQ1b antibody syndrome was first proposed by Odaka and Yuki in 2001. It is usually an autoimmune disease in which anti-GQ1b antibody is induced by microbial infection such as Campylobacter jejuni and Haemophilus influenzae, etc. and then GQ1b antibody combines with GQ1b antigens of trochlea, oculomotor, abducent nerve, brainstem and limb muscle spindle, resulting in central or peripheral nervous system lesions [5].At present, it is considered that the pathogenesis of anti-GQ1b antibody syndrome is a molecular simulation mechanism, and the infected microbial antigen has similar structure with some host antigen. After infection, autoantibodies or autoreactive T cells produced by cross-immunity attack specific ganglioside sites in peripheral and central nerves, resulting in nerve damage [6, 7].Gangliosides can be divided into GQ1b, GD1a, GT1a, GT1b and GM1 according to the position and number of hexose, among which GQ1b is mainly distributed in paraganglionic myelin sheath, neuromuscular junction and limb muscle spindle of cranial nerves III, IV, VI and VIII. Therefore, the clinical manifestations of anti-GQ1b antibody syndrome are mainly acute ophthalmoplegia, tendon reflex weakening or disappearance, and disturbance of consciousness [8, 9]. In addition to the above symptoms, atypical anti-GQ1b antibody syndrome can also have central or peripheral facial paralysis, ataxia, peripheral nerve sensory abnormality and distal limb fatigue [2]. Therefore, the clinical manifestations of anti-GQ1b antibody syndrome are various, and the positive serum anti-GQ1b IgG antibody is the diagnosis basis for this syndrome [1]. A history of antecedent infection within 4 weeks prior to the onset of neurological symptoms is an important medical history to support the diagnosis.

The clinical manifestation of this pediatric patient is mainly acute onset of vomiting. It is easy to be misdiagnosed as acute gastroenteritis in the first diagnosis, which did not improve after symptomatic treatment such as stomach protection and anti-vomiting, and later developed gait abnormalities and progressive worsening of symptoms, leading to the final inability to stand on their own. After asking the medical history, it was found that the pediatric patient had a history of precursor infection 6 days before the onset of the disease, and there was no obvious abnormality in improving blood biochemistry, cerebrospinal fluid routine, biochemistry and pressure, cephalometric CT, cranial contrast MRI, etc. Finally, the serum results reported that GQ1b antibody was positive, and then it was clearly diagnosed as anti-GQ1 antibody syndrome. According to previous reports, extraocular muscle paralysis is the most common initial sympotom in pediatric patients [4], and this pediatric patient started with vomiting followed by abnormal gait. To our knowledge, no similar cases have been reported so far. Combined with literature analysis of the possible causes of vomiting: ①Vomiting caused by increased intracranial pressure: The increased intracranial pressure may be caused by blocking subarachnoid space due to increased protein in cerebrospinal fluid [10]. In this case,the intracranial pressure is normal, and the protein in the cerebrospinal fluid was not significantly increased. This mechanism could not explain the vomiting occurred in this pediatric patient. ②Antibody-mediated effects on vestibular nerves: the GQ1b antigen is expressed in vestibular nerve, anti-GQ1b antibody binds to it, causing a vomiting response by involving vestibular structure, and most patients with vestibular involvement are accompanied by nystagmus and vertigo [11]. This pediatric patient has dizziness, but no vertigo, the presence of nystagmus was not found on multiple examinations, which is inconsistent with this mechanism. The cause of vomiting onset of phenotypic anti-GQ1b antibody syndrome needs further research to clarify. In addition, the abnormal gait appeared soon after vomiting, mainly in the early stage of the disease, which is characterized by a slight tilt of the upper body during walking as well as an opening and swaying of the legs at fast walking paces,and later progressing to inability to stand on his own. Physical examination shows normal muscle strength and muscular tension of all four limbs, finger-to-nose test, Romberg’s test (+), suspicious positive right Babinski’s sign. Combined with the EEG result of the pediatric patient that a small number of spike-wave-like waves emanating from the right parieto-occipital lobe were seen, antibody-mediated effect on cerebellum and cerebral cortex could be considered as a possible cause of abnormal gait.

Anti-GQ1b antibody syndrome can show the phenomenon of protein cell separation in cerebrospinal fluid and abnormal signal of cranial MRI, but the positive rate of examination is low. According to the previous reports, 7 pediatric patients with anti-GQ1b antibody syndrome showed a 28.6% positive rate of cerebrospinal fluid protein cell separation and 9 cases showed a 22% positive rate of abnormal cranial MRI signa [12];In 68 pediatric patients with anti-GQ1b antibody syndrome, the positive rate of cerebrospinal fluid protein cell separation was 34%, and the positive rate of abnormal signal on cranial MRI was only 18% [4]. In this case, no protein-cell separation was found in cerebrospinal fluid on two occasions, and no abnormality was found on two cranial contrast MRIs, which was consistent with the low positive rate reported previously, which makes the early diagnosis of anti-GQ1b antibody syndrome in pediatric patients more difficult.

Nerve electromyography is helpful to find out whether peripheral nerves are involved. According to a previous study with the largest number of cases and including 45 pediatric patients with anti-GQ1b antibody syndrome 60% of pediatric patients have abnormal peripheral nerve electromyography [4], suggesting that the incidence of peripheral nerve involvement is not low, but the reported 45 pediatric patients did not have the same phenotype as this case. The nerve electromyography in this pediatric patient suggested a slightly slow motor response in the upper extremities, but no abnormalities in peripheral nerve conduction in the extremities, so that this phenotype of anti-GQ1b antibody syndrome may not easily involve the peripheral nerves.

The prognosis of anti-GQ1b antibody syndrome is good. In this case, the child’s condition improved obviously after being treated with IVIG combined with large dose of methylprednisolone after definite diagnosis.However, due to the various clinical manifestations of anti-GQ1b antibody syndrome and clinicians have insufficient understanding of this rare disease,it is failed to diagnose it early, resulting in progressive aggravation of the child’s condition and excessive tests, which caused excessive psychological and economic burden to the child’s family. Therefore, clinicians should deepen their understanding of this kind of diseases, especially children who start with vomiting and later develop abnormal gait should be tested for relevant antibodies as soon as possible to clarify the diagnosis so as to give effective treatment in time.

In conclusion, the clinical manifestations of anti-GQ1b antibody syndrome are not specific. It usually needs to be considered as anti-GQ1b antibody syndrome after excluding other diseases with this as the main clinical manifestation. In contrast, atypical anti-GQ1b antibody syndrome, as a subtype of this disease, has a wide range of clinical manifestations, a variety of presentations and few case reports, making it easy to misdiagnose if there is insufficient understanding in clinical work. This study was conducted to deepen clinicians’ understanding of this rare disease through the report of atypical cases and literature review.

## Data Availability

Not applicable.

## Abbreviations

MRI: Magnetic resonance imaging
MRA: Magnetic Resonance angiography
DWI: Diffusion-weighted image
IVIG: Intravenous immunoglobulin
MFS: Miller-Fisher syndrome
AO: Acute ophthalmoparesis
BBE: Bickerstaff’s brainstem encephalitis
PCB: Pharynx-cervical-brachial weakness
EEG: Electro encephalograph
